# Hamartoma of the Vallecula in a Five-Month-Old Infant- A Case Report 

**Published:** 2019-05

**Authors:** Hiram Alvaréz-Neri, Stanislaw Sadowinski, Carlos de La Torre, Perla Villamor

**Affiliations:** 1 *Department of Pediatric Otolaryngology, Hospital Infantil de México, Federico Gómez, México.*; 2 *Department of Pathology, Hospital Infantil de México, Federico Gómez, México.*

**Keywords:** Hamartoma, Infancy, Pharyngeal Neoplasm

## Abstract

**Introduction:**

Hamartomas is a neoplasms composed of mature tissue elements from the affected site with disproportion between their components. Although lingual hamartomas are traditionally infrequent in the head and neck, a significant number of case reports with this disorder in infancy are arising from the literature.

**Case Report:**

We present a remarkable case of a vallecular hamartoma in a 5-month-old infant. Moreover, the value of histopathological diagnosis was highlighted regarding the differentiation between hamartomas and other benign/reactive lesions.

**Conclusion:**

Surgical excision is regarded as the treatment of choice for vallecular hamartomas; in addition, no recurrence has been reported after complete resection.

## Introduction

Hamartomas is a neoplasm composed of mature tissue elements from the affected site with a disproportion between its components (1,2). Its presence has been reported in all anatomical regions, with a higher incidence in the thoracic cavity and abdomen. Moreover, it has been reported in the nasosinusal area, nasopharynx, oral cavity, hypopharynx, esophagus, and larynx (2); however, it is infrequent in the head and neck regions. About 25 cases of lingual hamartomas have been reported in children.

 On the other hand, hamartomas of hypopharynx and larynx are extremely rare, with only 11 well-documented cases in the pediatric population until 1998 (2), and less than 10 cases until 2016 (3-8).The signs and symptoms of hamartomas vary according to its location. The most frequent symptoms are dysphagia and airway obstruction (cyanosis or apneas). Furthermore, 65% of cases present with stridor which involves the larynx (2). Despite its low incidence and variable presentation, the histopathological diagnosis of this disease is complex since this lesion is easily confused with different benign neoplasms (9,10). We present a remarkable case of an infant with dyspnea and intermittent stridor produced by a vallecular hamartoma, which was treated surgically with satisfactory results in the follow-up. 

## Case Report

**Fig 1:**
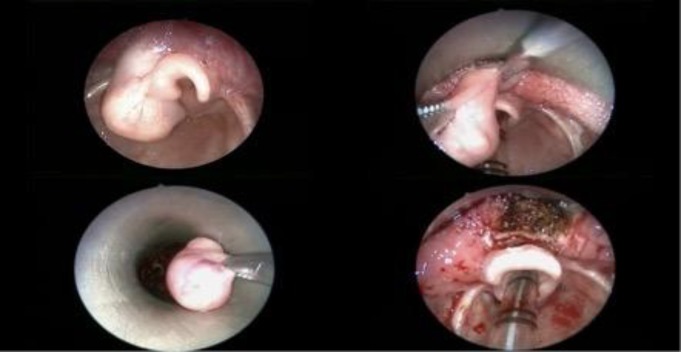
Sequence of microlaryngoscopic resection of the lesion. Endoscopic transoral excision of the lesion was performed through cold dissection plus electrocautery

A five-month-old male infant presented to the Department of Pediatrics with nocturnal coughs, dyspnea, episodic biphasic stridor with apneas, and intense drooling without dysphagia, three months before referral. On physical examination, using a tongue depressor during crying, the cystic appearance of a mass attached to the epiglottis emerged apparently. The results of a flexible laryngoscopy showed a rounded pink mass which was pedunculated in the vallecula. Moreover, the mass obstructed the airway at the supraglottic level and was mobile in synchrony with respiratory movements and crying. A non-contrast-enhanced computed tomography scan was performed and a smooth-edged homogeneous mass (approximately 1.5 cm in diameter) was detected in the vallecula. After the identification of a pedunculated mass in the vallecula, the patient underwent a direct laryngoscopy (Fig.1). Endoscopic transoral excision of the lesion was performed by cold dissection plus electrocautery (the FiO2 was previously decreased to 30% preventing the combustion of the airway, (Fig. 1). 

**Fig 2:**
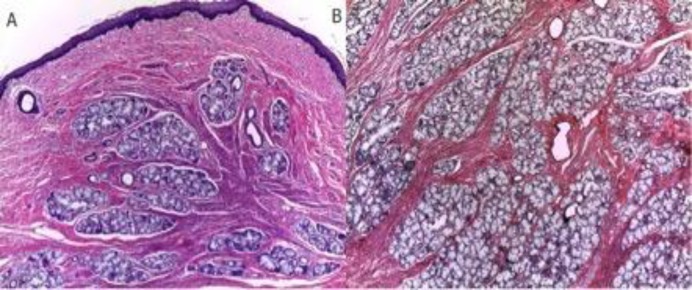
Histologic findings revealing a tumor with a mucous lining of normal characteristics (A), with abundant minor salivary glands, fat, and smooth muscle fibers distributed chaotically (B).

The patient remained intubated for 48 h, and he was subsequently extubated without complications and was prepared completely asymptomatic for discharge to home on day four after surgery. Histological evaluation showed fat and smooth muscle with a combination of nerves, vessels, and salivary glands, distributed in a disorganized manner (Fig 2, A,B). During postoperative follow-up, the patient has remained asymptomatic. An eight-month follow-up laryngoscopy was performed without persistence or recurrence of the mass.

## Discussion

Hamartomas are disorganized proliferation of normal tissues endogenous to their location. Although some genetic features during pregnancy could apparently have an impact on the etiology of hamartomas, the diagnosis still remains unclear (11). Hamartoma may appear in any organ; however, its location has been reported as infrequent in the head and neck. On the other hand, its incidence is higher in the first years of life (1,5,8). In the studies conducted by Krieger et al., despite their rare nature, hamartomas were represented as the third most frequent causes of childhood lingual lesions with a prevalence of 13.3%. In addition, they were followed by vascular/ lymphatic lesions (26.7%) and mucus extravasation phenomenon (16.3%) (10).

Clinically, the lesion is usually a mobile, polypoid mass with a pedicle that is covered by a smooth mucosa. Despite its low incidence, the early age of presentation and the morphological characteristics of the lesion generate a diagnostic suspicion. The differential diagnosis must include other benign tumors, especially vascular/lymphatic lesions (9). A contrast-enhanced radiological study, such as computed tomography, or magnetic resonance, can be useful to differentiate a vascularized lesion from an apparent hamartoma.

The histopathological evaluations showed similarities between the lesion and adjacent normal tissues. However, regarding cellular architecture, the cells were organized in unusual patterns. Furthermore, they were mainly composed of glandular structures, smooth muscle fibers, adipose tissues, and fibrous stroma, all covered by a normal squamous epithelium (1,9,10). 

With regard to lesion components, hamartomas are classified as mesenchymal or epithelial. Mesenchymal hamartomas are neoplasms containing tissues of mesodermal origin with no epithelial component. Pharyngeal hamartomas belong to epithelial hamartomas, containing fibrous tissue, thin muscle, cartilage, fat, skeletal tissue, epithelium, mucosa, salivary glands, and very occasionally neurogenic and vascular elements (1,6,9,10).

There are differential diagnoses between benign neoplasms and traumatic/reactive lesions (1,10). In hamartomas, neural and vascular components are usually entwined; however, in neuroma, the vascular and neural components are usually separated. Furthermore, fibrosis and swelling suggest reactive/traumatic etiology (1,10). The mentioned features were not evident in the present case.

The treatment of choice is surgical excision (2). Recurrences are associated with incomplete resection of the lesion; nevertheless, aggressive local recurrence after surgery has not been reported. Therefore, we suggest a complete resection of the tumor preserving the neighboring structures. In pharyngeal hamartomas, the transoral approach should be the primary surgical options in any of its modalities, such as cold dissection, laser, microdebrider, or radiofrequency ablation. However, the lesions with large size or those extending into deeper spaces merit an open approach. The prognosis after complete resection is excellent.

From a practical perspective, a hamartoma should be considered part of the differential diagnosis in cases of pharyngeal/lingual lesions causing obstruction in the airways in infants as in the present case. Contrast-enhanced imaging tools, such as computed tomography, or resonance magnetic imaging, should be used to complement the diagnostic approach (8). In cases of high diagnostic suspicion, an excisional biopsy may be indicated for diagnosis and management. The complete resection would be achieved; however, nonaggressive adjacent structures to the lesion can be ideally removed through minimally invasive techniques. Histopathologic characteristics will be the gold standard in the diagnosis of hamartomas. 

## Conclusion

Although this benign neoplasm is rare in the pharynx, it is a differential diagnosis to take into account in infants with a pharyngeal lesion. Lingual hamartomas in infants may not be considered as unusual as previously assumed. The importance of histopathology was highlighted in the differential diagnosis between hamartomas and other benign/ reactive lesions. The therapy of choice is surgical resection, and recurrence has not been reported after a complete resection surgery.
